# The relation between atherogenic index of plasma and cardiovascular outcomes in prediabetic individuals with unstable angina pectoris

**DOI:** 10.1186/s12902-023-01443-x

**Published:** 2023-08-31

**Authors:** Yang Liu, Xunxun Feng, Jiaqi Yang, Guangyao Zhai, Bin Zhang, Qianyun Guo, Yujie Zhou

**Affiliations:** 1 Beijing Key Laboratory of Precision Medicine of Coronary Atherosclerotic Disease, Clinical Center for Coronary Heart Disease, Department of Cardiology, Beijing Anzhen Hospital, Beijing Institute of Heart Lung and Blood Vessel Disease, Capital Medical University, Beijing, China; 2 Department of Cardiology, Fuwai Hospital, National Center for Cardiovascular Disease, Chinese Academy of Medical Science and Peking Union Medical College, Beijing, China

**Keywords:** Atherogenic index of plasma, Prediabetes, Unstable angina pectoris, Cardiovascular outcomes, Risk factor

## Abstract

**Background:**

The atherogenic index of plasma (AIP) is a novel biomarker associated with atherosclerosis, and an important risk factor for atherosclerosis, but its relation with cardiovascular prognosis in prediabetic patients with unstable angina pectoris (UAP) is still uncertain.

**Methods:**

This study included 1096 prediabetic patients with UAP who were subjected to follow-up for a maximum of 30 months, with cardiac death, refractory angina, and non-fatal myocardial infarction (MI) being the primary cardiovascular endpoints.

**Results:**

A significantly increased AIP was observed for the group with primary cardiovascular endpoints. Kaplan–Meier curves corresponding to these endpoints revealed pronounced differences between these two AIP groups (Log-rank *P* < 0.001). Multivariate Cox proportional hazards analyses highlighted AIP as being independent related to this primary endpoint (HR 1.308, 95% CI: 1.213–1.412, *P* < 0.001). AIP addition to the baseline risk model improved the prediction of the primary endpoint (AUC: baseline model, 0.622, vs. baseline model + AIP, 0.739, *P* < 0.001).

**Conclusions:**

AIP could be used to predict cardiovascular events in prediabetic individuals with UAP.

## Introduction

In recent years, the significance of triglycerides (TG) in atherosclerotic cardiovascular disease and the associated clinical practice has been paid increasing attention [1, 2]. The relevance of the lipid ratio or atherogenic indices are widely reported [3], among which the atherogenic index of plasma (AIP) was first proposed in 2001 as a comprehensive lipid index based upon the logarithm of the TG to high-density lipoprotein cholesterol (HDL-C) ratio [4]. Since AIP is closely related to the cholesterol esterification rate, lipoprotein particle size, and residual lipoproteinemia, it is thought to represent a valuable biomarker of plasma atherogenicity [5, 6].

Diabetic dyslipidemia is associated with increases in the levels of TGs with a concomitant drop in HDL-C levels without any corresponding changes in LDL-C levels. Notably, the LDL subfraction distribution in diabetic patients is shifted to small dense LDL (sdLDL), a subtype of LDL with smaller particles and higher density which is susceptible to multiple chemical modifications and further enhances atherosclerosis [7, 8]. As an inexpensive and easily assessed marker, AIP can be used to assess the progression of atherosclerosis, and studies have confirmed that AIP may become a useful substitute for sdLDL [9]. A subsequent study found that AIP was a powerful and reliable biomarker for predicting coronary artery disease (CAD) risk among individuals diagnosed with type 2 diabetes mellitus (T2DM) [10].

Prediabetes is defined by the elevation of blood glucose levels above the normal reference range but not at the threshold necessary for a diagnosis of T2DM, and it is generally considered an intermediate stage between T2DM and normoglycemia [11]. Atherogenic patterns corresponding to cardiovascular risk factors, including obesity, blood pressure, and dyslipidemia, are prominent in prediabetes patients prior to clinical T2DM development [12]. It has been found that the incidence of subclinical atherosclerosis in prediabetic subjects is significantly higher than that in normoglycemic subjects [13]. Considering that AIP is closely associated with elevated cardiovascular disease risk as compared with simple lipid levels [14], and the prognosis of AIP in prediabetic patients with unstable angina pectoris (UAP) is currently unclear, this study sought to investigate the association between AIP and cardiovascular prognosis in prediabetic individuals with UAP.

## Methods

### Study population

**Fig. 1:**
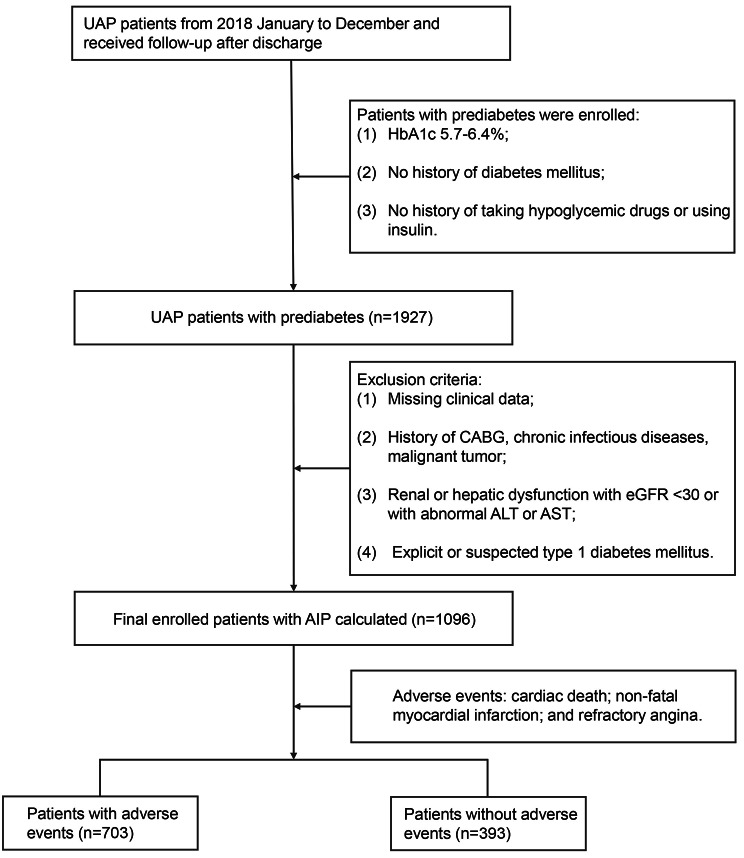
Flow chart of the study population enrollment. UAP: unstable angina pectoris; HbA1c: glycosylated hemoglobin A1c; CABG: coronary artery bypass grafting; eGFR: estimated glomerular filtration rate; ALT: alanine transaminase; AST: aspartate transaminase; AIP: atherogenic index of plasma

This was a single-center retrospective analysis, including 1927 UAP patients with prediabetes hospitalized in Beijing Anzhen Hospital between January and December of 2018. The exclusion criteria included: (1) severe hepatic insufficiency (abnormal aspartate aminotransferase or alanine aminotransferase) or renal failure insufficiency (estimated glomerular filtration rate (eGFR) < 30 ml/min/1.73 m^2^); (2) lack of clinical data; (3) history of cancer, coronary artery bypass grafting (CABG), or chronic infectious diseases; and (4) diagnosed or suspected to have a history of type 1 diabetes. Prediabetes [15] was defined as: (1) glycosylated hemoglobin A1c (HbA1c) at 5.7–6.4%; (2) no history of diabetes; and (3) no history of using hypoglycemic drugs or insulin therapy. The definition of UAP includes chest pain on exertion or rest within 2 weeks which are the presence of new or developing, in defect of elevated cardiac enzymes including high-sensitivity troponin I < 19.8 pg/ml and creatine kinase‐ MB < 6.3 ng/ml (the threshold of cardiac enzymes of Anzhen Hospital) [16, 17]. A total of 1096 prediabetic patients with UAP were finally included (Fig. 1).

### Data collection, definitions, and laboratory examination

Data included patient demographics and clinical characteristics (sex, age, systolic blood pressure (SBP), diastolic blood pressure (DBP), body mass index (BMI), smoking history, medical history, blood biomarkers, and pre-admission medication). The primary endpoint for this analysis consistent of events including cardiac death, refractory angina, and non-fatal myocardial infarction (MI). The secondary endpoints were the individual components of the primary endpoint. Patient follow-up was conducted by trained professional individuals via telephone interviews or outpatient clinic visits to collect relevant medical details at the 3, 6, and 12 month time points, and once per year thereafter for a maximum of 30 months. Before adjudicated by two experienced cardiologists, all events were verified via contact with the treating physicians and medical records. In addition, the mean follow-up time was 26.3 ± 6.5 months, with minimun 0.5 to maximun 30 months, and patients with the lower AIP levels were followed for a longer period (27.6 ± 4.8 vs. 24.1 ± 8.4, p < 0.001).

AIP was assessed from blood samples and calculated as follows: AIP = lg (TG/HDL-C), where each concentration is expressed in mmol/L [4, 18]. After fasting for 12 h, venous blood was collected from each patient. Biochemical parameters, including white TG, total cholesterol (TC), HDL-C, LDL-C, creatinine (Cr), eGFR, serum uric acid (SUA), fasting blood-glucose (FBG), C-reactive protein (CRP), and HbA1c, were analyzed using an automated biochemical analyzer in the clinical laboratory center of Beijing Anzhen Hospital. Ethics approval and consent to participateThe present study was approved by the Clinical Research Ethics Committee ofBeijing Anzhen Hospital, Capital Medical University, and all patients providedwritten informed consent for participation in the present study.

### Statistical analysis

Continuous variables were compared via one-way ANOVAs and Kruskal-Wallis tests, whereas categorical variable comparisons were made via chi-squared tests. Correlations of baseline characteristics were assessed via the Spearman or Pearson correlation tests. Receiver-operating characteristic (ROC) curve analyses were performed to establish optimal cutoff point values for AIP. Mainly based on clinical importance, the Cox proportional risk model was employed to evaluate the relations between AIP as a continuous variable and research results, including Model 1 (mainly including the demographic, physical and behavioral variables): adjusted for age, sex (male), BMI, SBP, DBP, smoking, hypertension, dyslipidemia, and prior percutaneous coronary intervention (PCI); Model 2 (based on Model 1 and added laboratory variables of lipids): additionally adjusted for TC and LDL-C, as well as the adjustments included in Model 1; Model 3 (based on Model 2 and added remaining laboratory variables): additionally adjusted for eGFR, FBG, HbA1c, Cr, CRP, and SUA as well as the adjustments included in Model 2; and Model 4 (based on Model 3 and added medication): additionally adjusted for statin usage, angiotensin converting enzyme inhibitor (ACEI), angiotensin receptor blocker (ARB), calcium channel blockers (CCB), β-blocker usage, and antiplatelet treatment, as well as the adjustments included in Model 3. The incremental value of AIP in the context of baseline risk model-based predictive analyses in a model considering risk factors such as age, sex (male), BMI, SBP, DBP, smoking, hypertension, dyslipidemia, prior PCI, TC, LDL-C, eGFR, Cr, CRP and SUA was assessed. Area under the curve (AUC) values for each model were compared via DeLong’s test. The Kaplan–Meier method was used to visualize the survival over time, and the survival curve was compared with the log-rank test. R 4.0.0 was used for all statistical analyses in this study, and in all analyses, *P* < 0.05 was considered statistically significant.

## Results

### Baseline characteristics

Under the grouping with or without cardiovascular adverse events, denoted as the event group and non-event group, respectively, there were 141 and 955 patients in the event and non-event groups, respectively. Average age and HDL-C values were significantly lower in the event group relative to the non-event group, whereas significant increases in BMI, TG, AIP, and SUA values for the event group were observed as compared to the non-event group, as shown in Table 1.

**Table 1:** Baseline clinical characteristics of patients with and without adverse event

	Total population (n = 1096)	Without event (n = 955)	With event (n = 141)	P value
Age, years	59.47 ± 9.86	59.77 ± 9.89	57.40 ± 9.45	0.008
Sex, male, n (%)	766 (69.9)	659 (69.0)	107 (75.9)	0.118
BMI, kg/m2	25.90 ± 3.44	25.79 ± 3.44	26.67 ± 3.38	0.004
SBP, mmHg	130.17 ± 17.17	130.34 ± 17.35	129.05 ± 15.92	0.407
DBP, mmHg	77.43 ± 10.84	77.37 ± 10.96	77.86 ± 10.03	0.615
Smoking, n (%)	528 (48.2)	458 (48)	70 (49.6)	0.776
Medical history, n (%)				
Hypertension	664 (60.6)	588 (51.6)	76 (53.9)	0.099
Dyslipidemia	784 (71.5)	690 (72.3)	94 (66.7)	0.203
Prior PCI	295 (26.9)	251 (26.3)	44 (31.2)	0.259
Laboratory results				
TG, mmol/L	1.53 ± 1.12	1.44 ± 0.97	2.12 ± 1.73	< 0.001
TC, mmol/L	4.11 ± 1.04	4.09 ± 1.02	4.20 ± 1.21	0.227
LDL-C, mmol/L	2.41 ± 0.89	2.40 ± 0.87	2.48 ± 1.00	0.353
HDL-C, mmol/L	1.17 ± 0.27	1.18 ± 0.28	1.06 ± 0.23	< 0.001
AIP	0.06 ± 0.28	0.04 ± 0.27	0.25 ± 0.26	< 0.001
CRP, mg/L	2.41 ± 4.33	2.38 ± 4.27	2.60 ± 4.73	0.581
Cr, mg/dL	71.25 ± 14.98	71.16 ± 15.05	71.90 ± 14.50	0.583
eGFR, mL/(min * 1.73 m2	95.43 ± 12.79	95.24 ± 12.76	96.71 ± 13.00	0.203
SUA, µmol/L	350.84 ± 86.07	348.71 ± 85.47	365.23 ± 88.99	0.033
FBG, mmol/L	5.93 ± 1.37	5.92 ± 1.37	5.97 ± 1.37	0.680
HbA1c, %	5.99 ± 0.22	5.99 ± 0.22	5.96 ± 0.21	0.067
Pre-admission medication, n (%)				
Antiplatelet therapy	1094 (99.8)	953 (99.8)	141 (100.0)	1
Statins	1091 (99.5)	950 (99.5)	141 (100.0)	0.848
ACEI	148 (13.5)	125 (13.1)	23 (16.3)	0.361
ARB	802 (73.2)	688 (72.0)	114 (80.9)	0.036
nitrate medication	1011 (92.2)	883 (92.5)	128 (90.8)	0.598
β-blocker	878 (80.1)	759 (79.5)	119 (84.4)	0.210
Angiographic data	Total population (n = 427)	Without event (n = 366)	With event (n = 61)	
Target vessel territory, n (%)				
LM	33 (7.7)	31 (8.5)	2 (3.3)	0.251
LAD	338 (79.2)	291 (79.5)	47 (77.0)	0.789
LCX	230 (53.9)	199 (54.5)	31 (50.8)	0.707
RCA	254 (59.5)	212 (57.9)	42 (68.9)	0.142
SYNTAX score	12.74 ± 7.55	12.92 ± 7.61	11.66 ± 7.19	0.229

BMI: body mass index; SBP: systolic blood pressure; DBP: diastolic blood pressure; PCI: percutaneous coronary intervention; TG: triglyceride; TC: total cholesterol; LDL-C: low density lipoprotein cholesterol; HDL-C: high density lipoprotein cholesterol; AIP: atherogenic index of plasma; CRP: C-reactive protein; Cr: creatinine; eGFR: estimated glomerular filtration rate; SUA: serum uric acid; FBG: fasting blood-glucose; HbA1c: glycosylated hemoglobin A1c; ACEI: angiotensin converting enzyme inhibitor; ARB: angiotensin receptor blocker; LM: left main artery; LAD: left anterior descending artery; LCX: left circumflex artery; RCA: right coronary artery; SYNTAX: synergy between PCI with taxus and cardiac surgery

After grouping AIP by the best cutoff value of the ROC curve, the high-AIP group included 393 patients and the low-AIP group included 703 patients. The age and HDL-C of the high-AIP group were significantly reduced as compared to the low-AIP group. The BMI, smoking percentage, TG, TC, LDL-C, AIP, Cr, SUA, FBG,β-blocker, and target vessel of right coronary artery usage were significantly increased in the high-AIP group. These results are displayed in Table 2.

**Table 2:** Baseline clinical characteristics of patients stratified by the optimal cutoff point of AIP

	Total population (n = 1096)	Lower AIP (< 0.134; n = 703)	Higher AIP (≥ 0.134; n = 393)	P value
Age, years	59.47 ± 9.86	60.75 ± 9.48	57.17 ± 10.12	< 0.001
Sex, male, n (%)	766 (69.9)	469 (66.7)	297 (75.6)	0.003
BMI, kg/m2	25.90 ± 3.44	25.33 ± 3.44	26.93 ± 3.20	< 0.001
SBP, mmHg	130.17 ± 17.17	130.58 ± 17.45	129.43 ± 16.67	0.287
DBP, mmHg	77.43 ± 10.84	77.27 ± 11.05	77.72 ± 10.47	0.511
Smoking, n (%)	528 (48.2)	309 (44.0)	219 (55.7)	< 0.001
Medical history, n (%)				
Hypertension	664 (60.6)	419 (59.6)	245 (62.3)	0.409
Dyslipidemia	784 (71.5)	500 (71.1)	284 (72.3)	0.740
Prior PCI	295 (26.9)	187 (26.6)	108 (27.5)	0.807
Laboratory results				
TG, mmol/L	1.53 ± 1.12	1.04 ± 0.33	2.42 ± 1.45	< 0.001
TC, mmol/L	4.11 ± 1.04	4.00 ± 1.04	4.30 ± 1.02	< 0.001
LDL-C, mmol/L	2.41 ± 0.89	2.34 ± 0.90	2.54 ± 0.86	< 0.001
HDL-C, mmol/L	1.17 ± 0.27	1.26 ± 0.27	1.00 ± 0.18	< 0.001
AIP	0.06 ± 0.28	-0.10 ± 0.17	0.35 ± 0.18	< 0.001
CRP, mg/L	2.41 ± 4.33	2.26 ± 4.46	2.69 ± 4.07	0.111
Cr, mg/dL	71.25 ± 14.98	69.58 ± 13.73	74.24 ± 16.59	< 0.001
eGFR, mL/(min * 1.73 m2	95.43 ± 12.79	95.55 ± 11.65	95.21 ± 14.63	0.672
SUA, µmol/L	350.84 ± 86.07	335.02 ± 80.60	379.12 ± 88.36	< 0.001
FBG, mmol/L	5.93 ± 1.37	5.81 ± 1.27	6.13 ± 1.52	< 0.001
HbA1c, %	5.99 ± 0.22	5.99 ± 0.22	5.99 ± 0.21	0.981
Pre-admission medication, n (%)				
Antiplatelet therapy	1094 (99.8)	702 (99.9)	392 (99.7)	1
Statins	1091 (99.5)	699 (99.4)	392 (99.7)	0.784
ACEI	148 (13.5)	89 (12.7)	59 (15.0)	0.317
ARB	802 (73.2)	517 (73.5)	285 (72.5)	0.768
nitrate medication	1011 (92.2)	645 (91.7)	366 (93.1)	0.483
β-blocker	878 (80.1)	539 (76.7)	339 (86.3)	< 0.001
Angiographic data	Total population (n = 427)	Lower AIP (< 0.134; n = 271)	Higher AIP (≥ 0.134; n = 156)	
Target vessel territory, n (%)				
LM	33 (7.7)	23 (8.5)	10 (6.4)	0.558
LAD	338 (79.2)	223 (82.3)	115 (73.7)	0.048
LCX	230 (53.9)	145 (53.5)	85 (54.5)	0.924
RCA	254 (59.5)	147 (54.2)	107 (68.6)	0.005
SYNTAX score	12.74 ± 7.55	13.04 ± 7.70	12.23 ± 7.28	0.289

BMI: body mass index; SBP: systolic blood pressure; DBP: diastolic blood pressure; PCI: percutaneous coronary intervention; TG: triglyceride; TC: total cholesterol; LDL-C: low density lipoprotein cholesterol; HDL-C: high density lipoprotein cholesterol; AIP: atherogenic index of plasma; CRP: C-reactive protein; Cr: creatinine; eGFR: estimated glomerular filtration rate; SUA: serum uric acid; FBG: fasting blood-glucose; HbA1c: glycosylated hemoglobin A1c; ACEI: angiotensin converting enzyme inhibitor; ARB: angiotensin receptor blocker; LM: left main artery; LAD: left anterior descending artery; LCX: left circumflex artery; RCA: right coronary artery; SYNTAX: synergy between PCI with taxus and cardiac surgery

### Correlations between AIP and cardiovascular risk factors

**Fig. 2:**
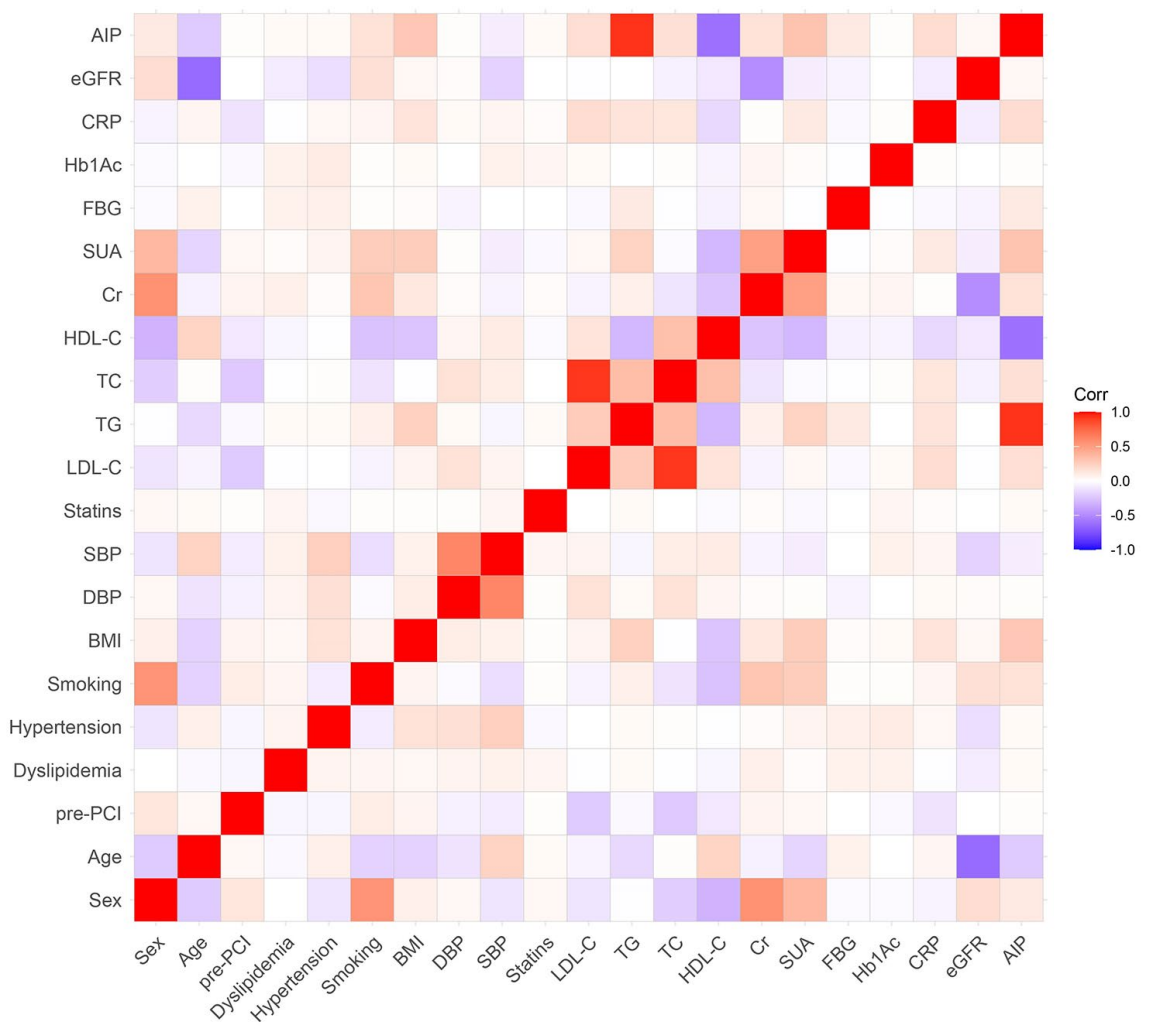
Correlations between the AIP and other factors. AIP: atherogenic index of plasma; eGFR: estimated glomerular filtration rate; CRP: C-reactive protein; HbA1c: glycosylated hemoglobin A1c; FBG: fasting blood-glucose; SUA: serum uric acid; Cr: creatinine; HDL-C: high density lipoprotein cholesterol; TC: total cholesterol; TG: triglyceride; LDL-C: low density lipoprotein cholesterol; SBP: systolic blood pressure; DBP: diastolic blood pressure; BMI: body mass index; PCI: percutaneous coronary intervention

Through correlation analyses, presented in Fig. 2, we found that AIP was positively associated with eGFR, LDL-C, TC, HT, DBP, hyperlipidemia, FBG, CRP, BMI, TG, HbA1c, statin usage, pre-PCI, smoking, SUA, sex, and Cr. In addition, AIP was significantly positively correlated with eGFR, HT, DBP, hyperlipidemia, statin usage, HbA1c, and pre-PCI. In contrast, AIP was inversely correlated with age and HDL-C, and no significant difference was found.

### Cardiovascular outcomes and Kaplan–Meier analysis

**Fig. 3:**
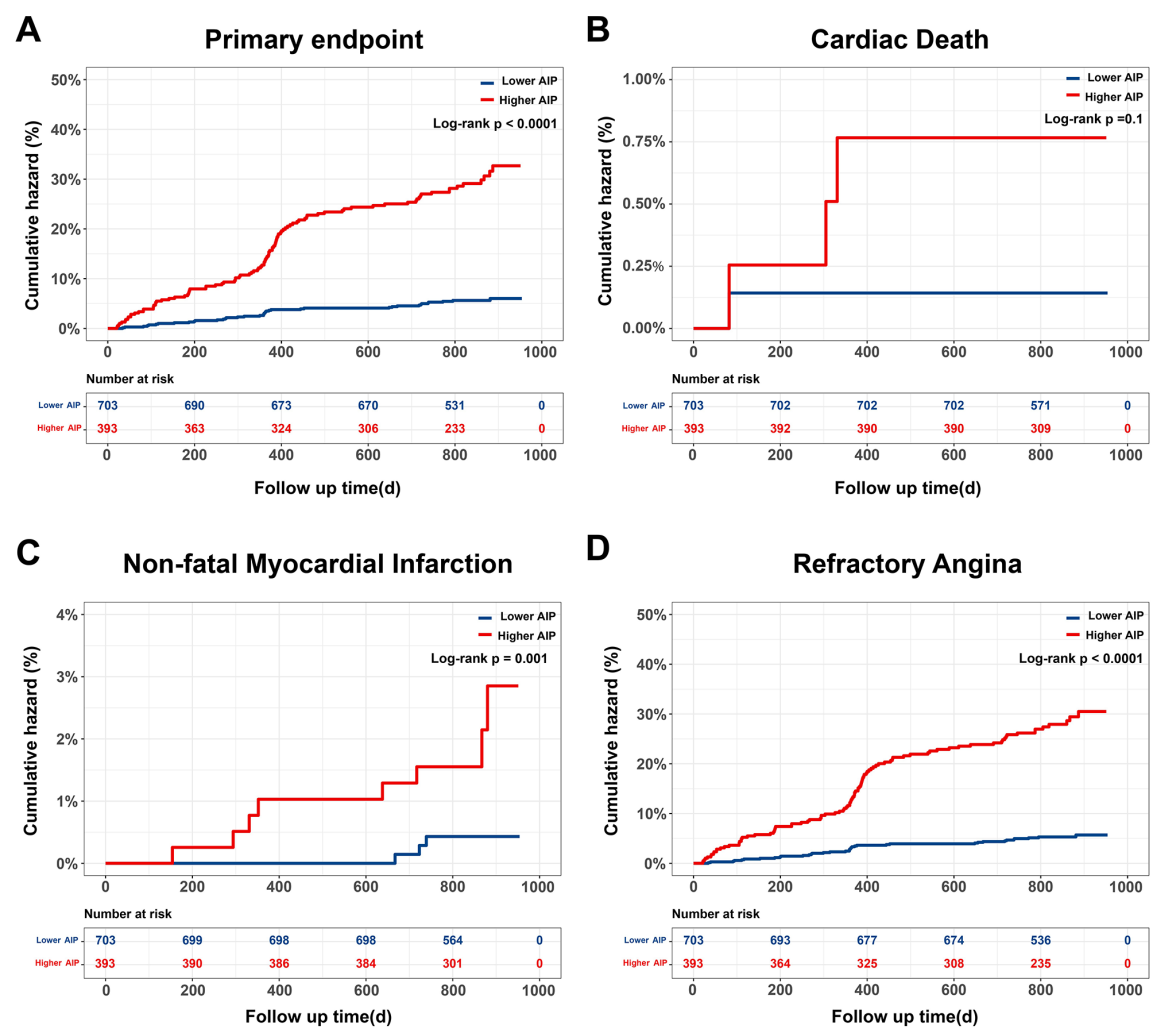
Kaplan–Meier curves for endpoint events according to the optimal cutoff point of AIP. A: Kaplan–Meier curves for primary endpoint; B: Kaplan–Meier curves for cardiac death; C: Kaplan–Meier curves for non-fatal myocardial infarction; D: refractory angina

At 30 months follow-up, there were four cases of cardiac death (0.4%), 11 cases of nonfatal myocardial infarction, and 134 cases of recurrent angina (12.2%) (Table 3). The incidences of the primary endpoint, refractory angina, and non-fatal MI rose significantly among individuals with a higher AIP (*P* < 0.05), although similar cardiac death rates were observed between groups (Table 3). Kaplan-Meier curve analyses corresponding to the primary endpoint revealed significant differences between AIP groups (Log-rank *P* < 0.001) (Fig. 3A). This difference was primarily attributable to the higher incidences of non-fatal MI and refractory angina (Log-rank *P* < 0.01) (Fig. 3C and D). Kaplan–Meier curves for cardiac death did not differ between groups (Log-rank *P* = 0.1) (Fig. 3B).

**Table 3:** Incidence of endpoint events according to the optimal cutoff point of AIP

	Total population (n = 1096)	Lower AIP (< 0.134; n = 703)	Higher AIP (≥ 0.134; n = 393)	P value
Primary endpoint, n (%)	141 (12.9)	39 (5.5)	102 (26.0)	< 0.001
Cardiac death, n (%)	4 (0.4)	1 (0.1)	3 (0.8)	0.266
Non-fatal MI, n (%)	11 (1.0)	3 (0.4)	8 (2.0)	0.025
Refractory angina, n (%)	134 (12.2)	37 (5.3)	97 (24.7)	< 0.001

AIP: atherogenic index of plasma; MI: myocardial infarction

### Evaluation of AIP in prognostic implication

In multivariate Cox proportional hazards analysis, we found that in all four models, after adjusting other influencing factors, AIP could be used as a predictor of the primary endpoint. These analyses revealed that each unit increase in AIP was independently related to increased primary endpoint risk (Model 1: HR 1.242, 95% CI 1.178–1.309, *P* < 0.001; Model 2: HR 1.303, 95% CI 1.212–1.400, *P* < 0.001; Model 3: HR 1.319, 95% CI 1.222–1.423, *P* < 0.001; Model 4: HR 1.308, 95% CI 1.213–1.412, *P* < 0.001) (Table 4). After multivariate analysis of model 4 with AIP for different outcomes, AIP was independently related to an elevated risk of non-fatal MI and refractory angina (non-fatal MI: HR 1.619, 95% CI 1.129–2.323, *P* = 0.009; refractory angina: HR 1.313, 95% CI 1.215–1.419, *P* < 0.001) (Table 5).

**Table 4:** Predictive value of AIP for primary endpoint in different Cox proportional hazards models

	HR	95% CI	P value
Model 1	1.242	1.178–1.309	< 0.001
Model 2	1.303	1.212-1.400	< 0.001
Model 3	1.319	1.222–1.423	< 0.001
Model 4	1.308	1.213–1.412	< 0.001

AIP: atherogenic index of plasma; HR: hazard ratio, CI: confidence interval

Model 1: adjusted for age, sex (male), BMI, SBP, DBP, smoking, hypertension, dyslipidemia, prior PCI

Model 2: adjusted for variables included in Model 1 and TC, LDL-C

Model 3: adjusted for variables included in Model 2 and eGFR, FBG, HbA1c, Cr, CRP, SUA

Model 4: adjusted for variables included in Model 3 and medication of statins, ACEI, ARB, CCB, β-blocker, antiplatelet

HR hazard ratio, CI confidence interval

**Table 5:** Predictive value of AIP for primary endpoint and each component in univariate and multivariate analysis

	Univariate analysis				Multivariate analysis		
	HR	95% CI	P value		HR	95% CI	P value
Primary endpoint	1.239	1.180–1.301	< 0.001		1.308	1.213–1.412	< 0.001
Cardiac death	1.261	0.948–1.679	0.112		1.213	0.727–2.022	0.460
Non-fatal MI	1.292	1.095–1.525	0.002		1.619	1.129–2.323	0.009
Refractory angina	1.236	1.175-1.300	< 0.001		1.313	1.215–1.419	< 0.001

AIP: atherogenic index of plasma; MI: myocardial infarction; HR: hazard ratio, CI: confidence interval. Multivariate analysis including age, sex (male), BMI, SBP, DBP, smoking, hypertension, dyslipidemia, prior PCI, TC, LDL-C, eGFR, FBG, HbA1c, Cr, CRP, SUA, medication of statins, ACEI, ARB, CCB, β-blocker, antiplatelet

### The incremental effects of AIP on adverse prognosis predictive value

**Fig. 4:**
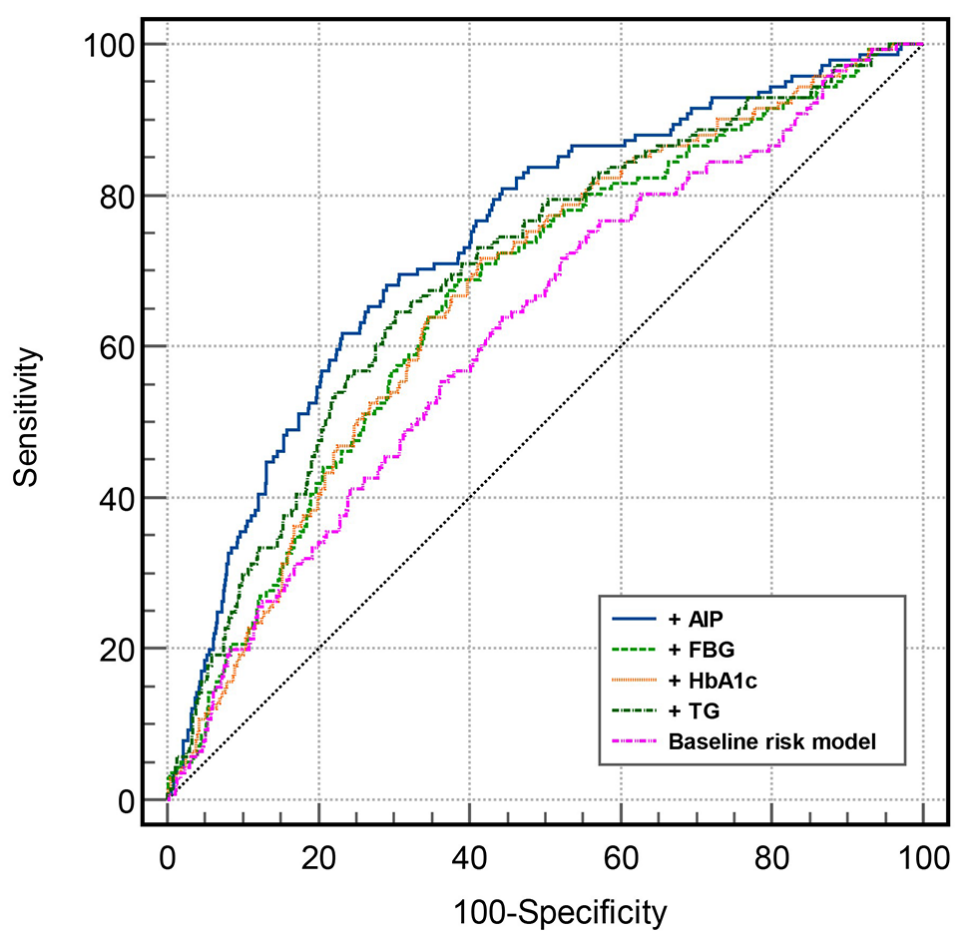
C-statistics evaluating incremental effect of FBG, HbA1c, TG and AIP beyond baseline risk model. AIP: atherogenic index of plasma; FBG: fasting blood-glucose; HbA1c: glycosylated hemoglobin A1c; TG: triglyceride

Adding AIP to the baseline risk model had a more significant incremental effect on the AUC obtained from the baseline risk model compared to adding FBG, HbA1c, or TG to the baseline risk model (AUC: baseline risk model, 0.622, vs. baseline risk model + AIP, 0.739, P < 0.001) (Table 6; Fig. 4).

**Table 6:** C-statistics for different risk models

	AUC	95% CI	P value	Z value	P for comparison
Baseline risk model	0.622	0.593–0.651	< 0.001	Reference	Reference
+ FBG	0.668	0.640–0.696	< 0.001	2.050	0.040
+ HbA1c	0.672	0.644-0.700	< 0.001	2.216	0.027
+ TG	0.700	0.672–0.727	< 0.001	3.451	< 0.001
+ AIP	0.739	0.712–0.764	< 0.001	4.718	< 0.001

AUC: area under the curve; FBG: fasting blood-glucose; HbA1c: glycosylated hemoglobin A1c; TG: triglyceride; AIP: atherogenic index of plasma. The baseline risk model includes age, sex (male), BMI, SBP, DBP, smoking, hypertension, dyslipidemia, prior PCI, TC, LDL-C, eGFR, Cr, CRP and SUA.

## Discussion

### AIP in metabolism and T2DM

In studies examining the relationship between AIP and cardiovascular health (CVH) scores, the number of desirable CVH markers was inversely related to the high prevalence of AIP [19]. Besides, moderate- and high-intensity physical activity are significantly negatively corelated with AIP [20]. In addition, it has been found that more aggregated risk factors in T2DM patients include increased TG, higher BP, central obesity, and insulin resistance. Potential mechanisms are increases in inflammation, oxidative stress, and endothelial cell dysfunction, which is associated with lower HDL-C levels [21]. AIP is a convenient clinical indicator that can aid in detecting high-risk T2DM complications and those of associated diseases, and it can be reliably used to monitor follow-up outcomes in high-risk T2DM patient populations [22]. One study found that the AIP of T2DM patients with metabolic syndrome was higher than that of T2DM patients without metabolic syndrome [23]. Through multivariate logistic regression analysis, another study found AIP to be independently related to metabolic syndrome, suggesting that lipid ratio can be used to reliably predict the risk of metabolic syndrome [24]. A third study proposed a direct relationship between dietary fat mass, increased BMI, and AIP dyslipidemia [25]. In our study, it was found that AIP was positively correlated with hyperlipidemia, HbA1c, and BMI, and it might be associated with the metabolism of lipids and diabetes.

### The molecular mechanism of AIP in atherosclerosis

The ability of lipids to migrate under the intima is an important step in the development of atherosclerosis. Lipids and their lipoprotein components have been designated as mediators and markers of CAD, characterized by a high ratio of LDL-C to HDL-C and an elevated level of TG [26]. Some studies have suggested AIP as an alternative to sdLDL particles [4] and shown that it may be a robust predictor for the risk of cardiovascular disease and atherosclerosis [27]. The specific mechanism of AIP and atherosclerosis is still unclear, but sdLDL is strongly atherosclerotic. This may be because sdLDL particles are more readily exposed on the surface, so they can easily penetrate the vascular endothelium and bind to arterial wall glycoproteins. As a result, lipid deposits gradually develop and are transformed into foam cells. Furthermore, sdLDL is easily oxidized to oxidized LDL-C [28], which can in turn aggregate adhesion molecules and chemokines, driving monocytes to differentiate into macrophages [29]. In response to cholesterol, large quantities of foam cells will be generated, thus inducing atherosclerosis. Moreover, sdLDL can suppress antioxidant production, thus accelerating atherosclerosis [30]. Studies have shown that AIP is the best factor to determine the cholesterol esterification rate of HDL-C and is more useful than conventional lipid parameters [31], so AIP could be considered as an auxiliary tool for personal blood lipid profiles [32]. Although our study did not include the analysis of basic research and the data of sdLDL, AIP may still become an alternative compound lipid index for sdLDL, providing a certain reference value for clinicians. However, more studies are still needed to explore the specific mechanism of AIP.

### The relationship between AIP and cardiovascular outcomes

Dyslipidemia is a key risk factor associated with CAD. AIP is a new comprehensive lipid index that may be a powerful predictor of risk in CAD [33]. Previous studies have pointed out that AIP is significantly associated with coronary artery calcium (CAC) progression in patients without cardiovascular disease (CVD). Although AIP is not an independent predictor of CAC progression, it should be considered along with other traditional risk factors when assessing CVD risk [34]. In patients with baseline CAC levels, AIP was also significantly related to the progression of CAC, and researchers have indicated that AIP has predictive value for CAC progression in adults who are asymptomatic and do not have severe CAC at baseline [35]. Based on a series of coronary computed tomography angiography (CCTA) results, AIP was proved to be an independent predictor of rapid plaque progression [36].

Regarding the prognostic capability of AIP, studies have found that AIP is positively related to CVD risk and may be a better predictor of mortality than individual cholesterol risk factors [37]. In a 15-year cohort study, researchers found that AIP could independently predict CVD development and its associated mortality [38]. Considering the presence of subclinical atherosclerosis could not be underestimated according to previous studies [39], the association of AIP with advanced subclinical CAD is also higher than that of traditional risk factors [40]. As for diabetic patients, studies have found that AIP may be a powerful biomarker when monitoring cardiovascular event risk in T2DM patients [41]. In non-diabetic patients, higher AIP levels may predict the development of ischemic heart disease [42]. Higher level of AIP is significantly associated with the prevalence of prediabetes and T2DM [43]. Compared with people with normal blood glucose, the level of AIP in patients with prediabetes is significantly increased, and the risk of CVD occurs earlier [44]. At the same time, Mahat et al. found that prediabetes patients have abnormal AIP, suggesting that prediabetes is prone to develop CVD [45]. In addition, El-Eshmawy et al. have confirmed that AIP is significantly correlated with inflammatory indicators in prediabetes, which indicates that AIP may participate in atherosclerosis through inflammatory response [46]. POST-PCI study, a multicenter randomized clinical trial with a primary endpoint including hospitalization for UAP, included 80% of patients with angina [47]. In the concurrent ISCHEMIA trial, approximately 20% of patients had previously undergone PCI, which was similar to our study. Approximately two thirds of the patients in this trial had angina symptoms in the four weeks prior to randomization [48]. With reference to these two large clinical studies, we identified the composite endpoints in our study. In our study, through multivariate regression analysis, it was found that in model 4, AIP was independently and positively correlated with the primary endpoint, and the predictive value for adverse prognosis also suggested that AIP was better than other blood indicators based on the baseline risk model. We explored the relation between AIP and cardiovascular outcomes in prediabetic patients with UAP, and the results suggested that patients with higher levels of AIP may have more cardiovascular events. In conclusion, the study of AIP-related prognosis provides a potential direction for studying CVD risk in UAP and developing intervention strategies for these patients.

## Conclusion

As a composite lipid index, AIP is closely related to the prognosis of prediabetic patients with UAP, and it has the potential to be a convenient and valuable clinical reference index.

### Limitations

Our study has certain limitations. First, our study is a follow-up study, not a multi-center randomized controlled trial with a high level of evidence, so the conclusions drawn from the study may be biased. In addition, the validation procedure of this study is still on going. Second, AIP is an index calculated based on previous research results. The significance of our research is to provide a certain clinical reference value. AIP may represent the components of sdLDL, but its specific mechanism in the human body and the prognosis of patients is still unknown. More research on its mechanism is needed.

## Data Availability

The datasets used and analyzed during the current study are available from the corresponding author on reasonable request.

## Abbreviations

AIP: atherogenic index of plasma
UAP: unstable angina pectoris
TG: triglyceride
HDL-C: high density lipoprotein cholesterol
LDL-C: low density lipoprotein cholesterol
sdLDL: small dense low density lipoprotein
CAD: coronary artery disease
T2DM: type 2 diabetes mellitus
SAP: unstable angina pectoris
eGFR: estimated glomerular filtration rate
HbA1c: glycosylated hemoglobin A1c
CABG: coronary artery bypass grafting
BMI: body mass index
SBP: systolic blood pressure
DBP: diastolic blood pressure
MI: myocardial infarction
TC: total cholesterol
CRP: C-reactive protein
Cr: creatinine
SUA: serum uric acid
FBG: fasting blood-glucose
ROC: receiver-operating characteristic
PCI: percutaneous coronary intervention
ACEI: angiotensin converting enzyme inhibitor
ARB: angiotensin receptor blocker, CCB:calcium channel blockers
AUC: area under the curve
CVH: cardiovascular health
CAC: coronary artery calcium
CAD: cardiovascular disease
CCTA: coronary computed tomography angiography
